# Impact of health intervention coverage on reducing maternal mortality in 126 low- and middle-income countries: a Lives Saved Tool modelling study

**DOI:** 10.1186/s41256-025-00414-0

**Published:** 2025-04-02

**Authors:** Xi-Ru Guo, Jue Liu, Hai-Jun Wang

**Affiliations:** 1 Department of Maternal and Child Health, School of Public Health, Peking University, National Health Commission Key Laboratory of Reproductive Health, Peking University Health Science Center-Weifang Joint Research Center for Maternal and Child Health, No. 38 Xueyuan Rd, Haidian District, Beijing, 100191 China; 2 Department of Epidemiology and Biostatistics, School of Public Health, Peking University, No. 38 Xueyuan Rd, Haidian District, Beijing, 100191 China

**Keywords:** Maternal mortality, Health intervention, Low- and middle-income countries, Modelling study

## Abstract

**Background:**

There is a continued and urgent need to address the stagnation of the global maternal mortality ratio (MMR), especially for low- and middle-income countries (LMICs). We aimed to assess the impact of scaling up health intervention coverage on reducing MMR under four scenarios for 126 LMICs.

**Methods:**

We conducted the modelling study to estimate MMR and additional maternal lives saved by intervention by 2030 for 126 LMICs using the Lives Saved Tool (LiST). We applied four scenarios to assess the impact of scaling up health intervention coverage with no scale-up (no change), a modest scale-up (increased by 2% per year), a substantial scale-up (increased by 5% per year), and universal coverage (coverage reached 95% by 2030). In sensitivity analysis, with the current trend, we assumed that coverage of each intervention remained unchanged from 2024, with MMR changing according to the annual percentage change (APC) of 2015–2020.

**Results:**

Among the 126 LMICs, 31.7% (40/126) countries showed an increase in MMR, and 38.1% (48/126) stalled on the reduction of MMR from 2015 to 2020. With a modest, substantial, or universal scale-up, the 2030 average MMR would be 172.1 (117.6–262.9), 139.8 (95.6–213.5) or 98.6 (67.8–149.7), not reaching the SDG Target 3.1. Additional maternal lives saved by scaling up the coverage of health interventions were mainly clustered in the African Region, the Southeast Asia Region, and the Eastern Mediterranean Region. Compared with other included interventions, uterotonics for postpartum hemorrhage, assisted vaginal delivery and cesarean delivery played more important roles in reducing maternal mortality.

**Conclusions:**

The study findings highlighted that even under a substantial scale-up of intervention coverage or scaling up to universal coverage of interventions, it would be difficult for the 126 LMICs to achieve the SDG Target 3.1 on time. Optimizing the allocation of health resources, promoting health equality, taking more decisive national, regional and global actions are urgently needed for LMICs to reduce MMR and reach the SDG Target 3.1.

**Supplementary Information:**

The online version contains supplementary material available at 10.1186/s41256-025-00414-0.

## Background

Advancing women's health and well-being is one of the focuses for achieving Sustainable Development Goals (SDGs) [1]. According to MMR estimates for 2020 by the World Health Organization (WHO), United Nations International Children's Emergency Fund (UNICEF), United Nations Population Fund (UNFPA), World Bank Group, and United Nations Department of Economic and Social Affairs (UNDESA)/Population Division, approximately 287,000 women died during and following pregnancy and childbirth in 2020 in the world and 95% of maternal deaths occurred in low- and middle-income countries (LMICs) in 2020 [2]. Although substantial progress has been made in reducing global maternal mortality in the past two decades, further efforts are needed for LMICs to reduce preventable maternal deaths.

Accelerated actions are urgently required to address stagnating MMR globally and reach the SDG Target 3.1 on time [3]. Scaling up the coverage of maternal health interventions is one of the most effective ways to reduce and eliminate preventable maternal mortality [4]. However, most maternal health interventions have not yet achieved universal coverage in LMICs [5]. Quantifying the effect of health intervention coverage on reducing maternal mortality at national, regional and global levels could provide scientific evidence for optimizing the priority of health system investment in maternal health interventions and services. One national study using data from 2015 and before, assessed the impact of maternal health interventions on maternal mortality in Mozambique [6]. A regional research focusing on ASEAN (the Association of Southeast Asian Nations) illustrated the effect of intervention coverage on maternal mortality, applying data from 1990 to 2008 [7]. One global study using data in 2017 showed the potential impact of midwives on maternal mortality in LMICs [8]. However, there is a lack of related studies in LMICs across the six WHO regions that both use latest data after the COVID-19 and include full-period maternal health interventions.

This study estimates the impact of scaling up full-period maternal health interventions on maternal mortality in the 126 LMICs to provide actionable insights for achieving the SDG Target 3.1. We estimated MMR and additional maternal lives saved by intervention under various scenarios for scaling up coverage of health interventions by 2030. Findings from this study will be beneficial for health planners among LMICs to optimize the allocation of health resources, promote health equality, take more decisive national, regional and global actions.

## Methods

### Study design

Our modelling study involved three phases: (i) showing MMR estimates in 2000–2020 and detecting potential points when a change in the linear slope of the trend of MMR happened among the 126 LMICs (Table S1); (ii) describing percent of maternal deaths in 2021 by cause among six WHO regions; (iii) estimating MMR and additional maternal lives saved by intervention under various scenarios for scaling up health intervention coverage by 2030.

Based on a systematic analysis of global causes of maternal deaths, we categorized maternal hemorrhage into antepartum hemorrhage, intrapartum hemorrhage, and postpartum hemorrhage. Among maternal hemorrhage, antepartum hemorrhage comprised 24%, intrapartum hemorrhage comprised 3%, and postpartum hemorrhage comprised 73% [9]. The causes of maternal deaths in this study included antepartum hemorrhage, intrapartum hemorrhage, postpartum hemorrhage, hypertensive disorders, sepsis, abortion, other direct causes, and indirect causes.

We included fourteen maternal health interventions, which both had proven efficacy, known effectiveness values and complete data, containing one periconceptual intervention, three pregnancy interventions, and ten childbirth interventions. Periconceptual intervention was safe abortion services. Pregnancy interventions were TT—Tetanus toxoid vaccination, micronutrient supplementation (iron and multiple micronutrients) and hypertensive disorder case management. Childbirth interventions were clean birth environment, MgSO_4_ for eclampsia, antibiotics for preterm or prolonged premature rupture of membranes (PROM), antibiotics for maternal sepsis, assisted vaginal delivery, uterotonics for postpartum hemorrhage, manual removal of placenta, removal of retained products of conception, cesarean delivery and blood transfusion.

Based on previous studies [8], to meet the needs of different countries and regions, our analysis presented the impacts of scaling up health intervention coverage in four scenarios. With no scale-up (Scenario 0), we assumed that coverage of each intervention would remain unchanged from 2024. With a modest scale-up (Scenario 1), we assumed that coverage of each intervention increased by 2% annually up to a maximum of 100%. With a substantial scale-up (Scenario 2), we assumed that coverage of each intervention increased by 5% annually up to a maximum of 100%. With universal coverage (Scenario 3), we assumed that the coverage of each intervention would reach 95% by 2030 (Table 1).

**Table 1:** Scenarios used to model the impact of health interventions on maternal mortality

	Description	Percentage change in intervention coverage rates
0	No scale-up	No change from baseline (2024) coverage rates
1	Modest scale-up	2% increase in baseline coverage rates up to a maximum of 100%
2	Substantial scale-up	5% increase in baseline coverage rates up to a maximum of 100%
3	Universal coverage	95% coverage by 2030

### Tools and data sources

In this modelling study, we used the Lives Saved Tool (LiST) of the Spectrum, version 6.36, software suite to estimate MMR and additional maternal lives saved for scaling up health intervention coverage by 2030. This software incorporated the latest available live birth number, maternal health intervention coverage, the efficacy and affected fraction of interventions on cause-specific maternal mortality [10] (Table S2). The baseline year of this study was 2024. The baseline MMR data was MMR estimates for 2020 from WHO, UNICEF, UNFPA, World Bank Group, and UNDESA/Population Division. The baseline proportion of maternal deaths was updated by using data on the causes of maternal deaths for women aged 15–49 years from Global Burden of Disease (GBD) 2021. Detailed methodologies and data sources utilized in GBD 2021 have been reported [11]. This model calculation formulas:

- Estimated MMR = MMR × (1 − Percent of maternal death by cause × Intervention coverage change × Efficacy × Affected fraction)
- Additional maternal lives saved = Live birth number × MMR × Percent of maternal death by cause × Intervention coverage change × Efficacy × Affected fraction

### Statistical analysis

According to previous studies [12, 13], to detect potential points when a change in the linear slope of the trend of MMR happened and to further determine whether MMR has stagnated or increased, we applied joinpoint regression models using data from 2000 to 2020 for every country among the 126 LMICs. An annual percentage change (APC) and the average APC (AAPC) were calculated in this study. The four scenarios we assumed were that the coverage of each intervention changed, with MMR remaining unchanged. Sensitivity analysis was conducted by estimating the MMR in 2030 from APC of 2015–2020. In sensitivity analysis, with the current trend (Scenario 0), we assumed that coverage of each intervention remained unchanged from 2024, with MMR changing according to the APC of 2015–2020.

Considering that the uncertainty inherent in maternal mortality ratio, we computed 80% uncertainty intervals (10th and 90th percentiles of the posterior distributions) of MMR in 2020 using the standard error of MMR. The data can be interpreted as meaning that there is an 80% chance that the true value lies within the *UI*, a 10% chance that the true value lies below the lower limit and a 10% chance that the true value lies above the upper limit [2]. Uncertainty intervals (80%*UI*) of all estimated MMR in 2030 were calculated from 80% *UI* of MMR in 2020.

## Results

### Maternal mortality and causes of death

The 2020 average MMR of the 126 LMICs was 200 maternal deaths per 100,000 live births. In 2020 the average MMR of LMICs in the African Region was 398, the average MMR of LMICs in the Region of the Americas was 87, the average MMR of LMICs in the Southeast Asia Region was 113, the average MMR of LMICs in the European Region was 17, the average MMR of LMICs in the Eastern Mediterranean Region was 156, and the average MMR of LMICs in the Western Pacific Region was 89 (Table 2).

**Table 2:** MMR and APC, AAPC of MMR in 2000–2020 by region among 126 LMICs

Regions	MMR					APC			AAPC
	2000	2005	2010	2015	2020	2000–2010	2010–2015	2015–2020	
Worldwide	354	296	262	223	200	− 3.74	− 3.26	− 1.47	− 2.97
African Region	670	566	518	444	398	− 2.87	− 2.44	− 2.70	− 2.64
Region of the Americas	119	103	90	84	87	− 3.06	− 2.25	1.93	− 1.48
Southeast Asia Region	315	245	191	147	113	− 5.31	− 4.48	− 3.97	− 4.58
European Region	38	29	22	18	17	− 5.94	− 6.17	− 0.79	− 4.84
Eastern Mediterranean Region	316	265	216	182	156	− 4.24	− 3.35	− 1.90	− 3.24
Western Pacific Region	175	142	117	96	89	− 3.15	− 2.90	− 1.25	− 2.57

Maternal mortality ratio (MMR) is defined as maternal deaths per 100,000 live births for women of reproductive age (15–49 years). APC was the annual percentage change,the, and AAPC was the average yearly percentage change calculated using joinpoint regression

Among all the included LMICs, 31.75% (40/126) of countries showed an increase in MMR. 38.10% (48/126) of countries have stalled of MMR since 2015 (the decline rate of MMR after 2015 is less than the decline rate before 2015) (Table S3). LMICs in the Region of the Americas showed an increase in MMR. LMICs in the Southeast Asia Region, the European Region, the Eastern Mediterranean Region, and the Western Pacific Region have stalled on the reduction of MMR since 2015. LMICs in the African Region showed a decline in MMR (Table 2).

Except for other direct and indirect causes, hypertensive disorders and postpartum hemorrhage were the leading causes of maternal deaths in the 126 LMICs. For LMICs among six regions, hypertensive disorders and postpartum hemorrhage accounted for 11.41–19.00% and 10.78–19.97%, respectively (Table S4).

### Estimated MMR and additional maternal lives saved by 2030

We estimated that relative to no scale-up, with a modest, substantial, or universal scale-up, the 2030 average MMR of the 126 LMICs would be 172.05 (80%*UI*: 117.62–262.87), 139.84 (80%*UI*: 95.60–213.52) or 98.59 (80%*UI*: 67.82–149.68). Relative to status in 2030 with the current trend, under a substantial scale-up of coverage or scaling up to universal coverage, the average MMR in 2030 of LMICs would avert 12.71%, 27.03%, or 43.33% of maternal deaths.

**Fig. 1:**
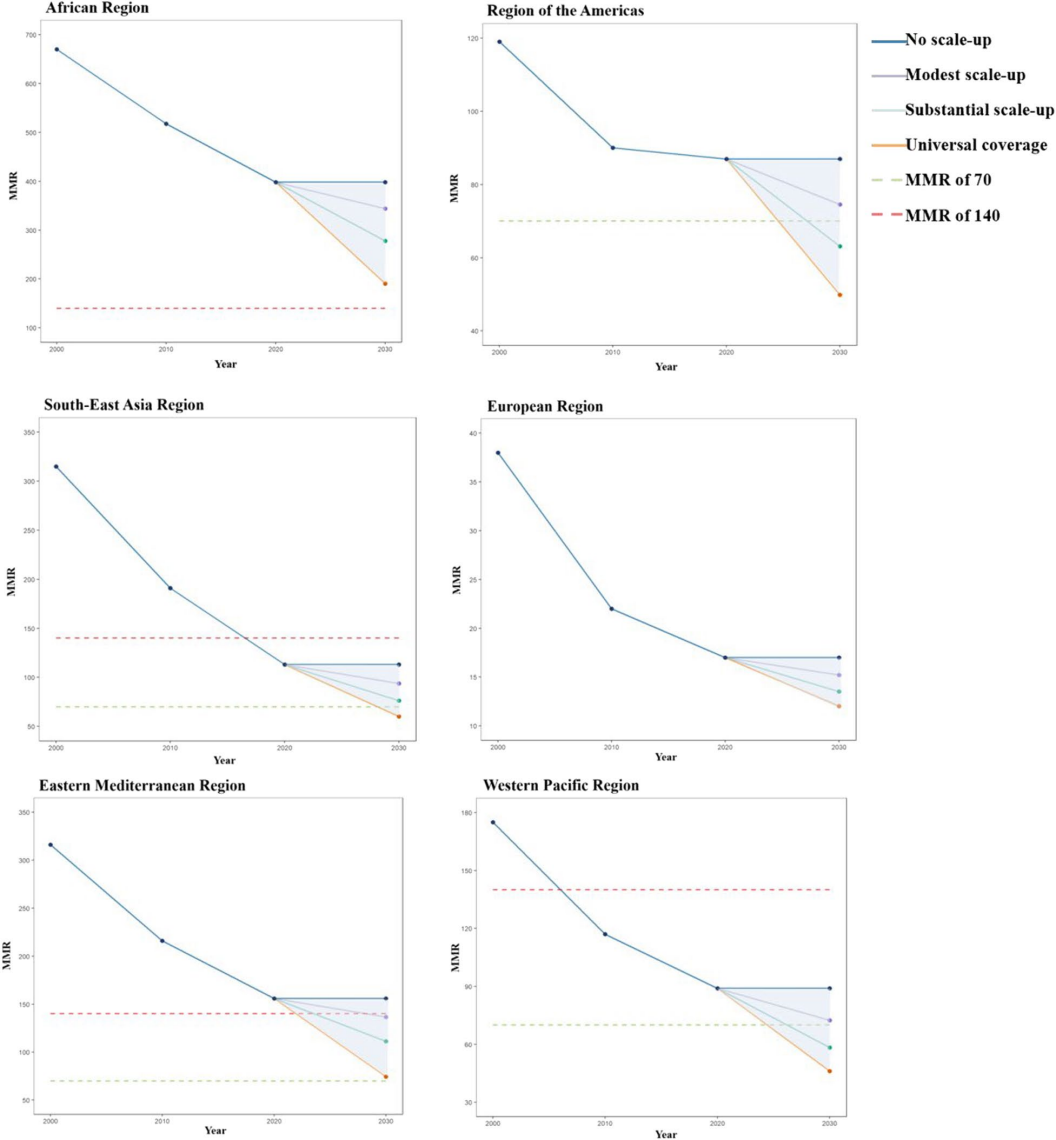
MMR under four scenarios among 126 LMICs in 2000–2030. *Notes*: Maternal mortality ratio (MMR) is defined as maternal deaths per 100,000 live births for women of reproductive age (15–49 years). Modest scale-up (Scenario 1): we assumed that coverage of every health intervention increased by 2% per year up to a maximum of 100%. Substantial scale-up (Scenario 2): we assumed that coverage of every health intervention increased by 5% per year to a maximum of 100%. Universal coverage (Scenario 3): we assumed that coverage of every health intervention would reach 95% by 2030

Under a modest scale-up, except for the European Region, the average MMR of LMICs in another five regions in 2030 would not achieve SDG Target 3.1. Under a substantial scale-up, the 2030 average MMR of LMICs in the Region of the Americas, the European Region, the Western Pacific Region could meet SDG Target 3.1. Under scaling up to universal coverage, the 2030 average MMR of LMICs in the Region of the Americas, the Southeast Asia Region, the European Region, the Western Pacific Region would reach the SDG Target 3.1 (Fig. 1).

Even under a substantial or universal scale-up by 2030, LMICs in the African Region would not reach the SDG Target 3.1 on time. Under a substantial or universal scale-up, the average MMR of LMICs in the Region of the Americas would be 63.09 or 49.83, reaching the SDG Target 3.1. Under scaling up to universal coverage, the average MMR of LMICs in the Southeast Asia Region would be 60.20. Under a modest, substantial or universal scale-up, the average MMR of lower-middle-income countries in the Eastern Mediterranean Region would be 64.55, 54.12, or 43.38. Under a substantial or universal scale-up, the average MMR of LMICs in the Western Pacific Region would be 58.35 or 46.15, meeting the SDG Target 3.1 (Table 3, Table S5).

**Table 3:** Estimated relative reductions of MMR by 2030 in four scenarios by region among 126 LMICs

	Scenario 0 (No scale-up)	Scenario 1 (Modest scale-up)		Scenario 2 (Substantial scale-up)		Scenario 3 (Universal coverage)	
	MMR	MMR (80%*UI*)	Reduction (%)	MMR (80%*UI*)	Reduction (%)	MMR (80%*UI*)	Reduction (%)
Worldwide	200	172.05 (117.62, 262.87)	13.81	139.84 (95.60, 213.52)	27.39	98.59 (67.82, 149.68)	42.53
Low-income countries	456	396.89 (263.17, 616.66)	13.39	318.66 (211.11, 495.22)	30.52	198.08 (132.46, 305.06)	55.04
Lower middle-income countries	202	170.89 (119.38, 258.71)	15.18	137.51 (96.00, 208.05)	30.78	103.07 (71.67, 156.14)	46.16
Upper middle-income countries	65	56.29 (40.17, 83.05)	12.65	49.19 (35.13, 72.50)	22.37	42.39 (30.34, 62.42)	32.40
African Region	398	343.87 (238.22, 517.01)	13.72	277.84 (192.41, 417.64)	29.75	190.58 (132.47, 285.23)	48.83
Low-income countries	501	435.59 (296.28, 660.19)	13.71	349.59 (237.78, 529.75)	31.02	218.10 (149.57, 327.60)	55.26
Lower middle-income countries	376	321.23 (229.23, 474.33)	14.26	259.56 (185.02, 383.11)	30.62	194.31 (137.84, 287.10)	47.18
Upper middle-income countries	161	140.05 (95.45, 217.66)	12.39	119.85 (81.77, 186.09)	23.91	102.36 (69.84, 159.37)	34.70
Region of the Americas	87	74.54 (56.31, 101.87)	12.64	63.09 (47.90, 85.57)	23.56	49.83 (38.33, 66.37)	36.84
Lower middle-income countries	165	136.88 (94.04, 211.54)	16.62	103.60 (71.29, 159.65)	33.91	64.47 (44.55, 98.61)	53.18
Upper middle-income countries	69	60.69 (47.93, 77.50)	11.76	54.09 (42.70, 69.11)	21.26	46.58 (36.95, 59.20)	33.20
South-East Asia Region	113	93.89 (66.21, 143.41)	15.37	76.37 (53.83, 116.58)	29.73	60.20 (42.75, 90.98)	43.76
Low-income countries	107	91.37 (39.28, 212.63)	14.61	75.36 (32.40, 175.37)	29.57	48.15 (20.70, 112.05)	55.00
Lower middle-income countries	125	104.61 (77.04, 150.41)	15.41	85.09 (62.63, 122.31)	30.51	67.13 (49.42, 96.55)	45.65
Upper middle-income countries	86	69.73 (49.91, 104.01)	15.54	56.36 (40.44, 83.61)	27.96	48.04 (34.53, 70.94)	35.60
European Region	17	15.22 (10.50, 22.52)	13.39	13.53 (9.35, 20.00)	22.90	12.03 (8.34, 17.80)	30.03
Lower middle-income countries	32	27.73 (19.78, 40.19)	14.66	23.05 (16.53, 33.25)	29.79	19.60 (14.06, 28.24)	40.90
Upper middle-income countries	14	12.54 (8.51, 18.73)	13.11	11.49 (7.82, 17.16)	21.43	10.41 (7.11, 15.56)	27.70
Eastern Mediterranean Region	156	136.55 (79.67, 237.52)	11.48	111.48 (64.73, 194.35)	24.59	74.46 (43.73, 129.14)	42.48
Low-income countries	345	303.23 (175.51, 523.34)	11.88	243.57 (140.18, 421.07)	28.69	147.96 (86.41, 253.51)	54.18
Lower middle-income countries	75	64.55 (39.05, 112.18)	11.79	54.12 (32.63, 94.26)	23.40	43.38 (25.97, 75.98)	37.20
Upper middle-income countries	57	50.78 (28.23, 95.40)	9.97	44.32 (24.60, 83.40)	20.93	34.86 (19.97, 63.60)	37.08
Western Pacific Region	89	72.50 (45.32, 123.61)	17.59	58.35 (36.44, 99.68)	32.26	46.15 (28.58, 79.64)	45.33
Lower middle-income countries	109	88.28 (55.57, 148.06)	18.98	69.13 (43.56, 115.80)	36.19	52.21 (32.85, 87.65)	50.62
Upper middle-income countries	49	40.94 (24.81, 74.69)	14.81	36.80 (22.19, 67.44)	24.38	34.03 (20.02, 63.61)	34.74

Maternal mortality ratio (MMR) is defined as maternal deaths per 100,000 live births for women of reproductive age (15–49 years). All estimates' uncertainty intervals (*UI*) refer to the 80% uncertainty intervals (10th and 90th percentiles of the posterior distributions). No scale-up (Scenario 0), we assumed that coverage of every health intervention change from baseline. Modest scale-up (Scenario 1): we assumed that coverage of every health intervention increased by 2% per year to a maximum of 100%. Substantial scale-up (Scenario 2): we assumed that coverage of every health intervention increased by 5% per year up to a maximum of 100%. Universal coverage (Scenario 3): we assumed that coverage of every health intervention would reach 95% by 2030

**Fig. 2:**
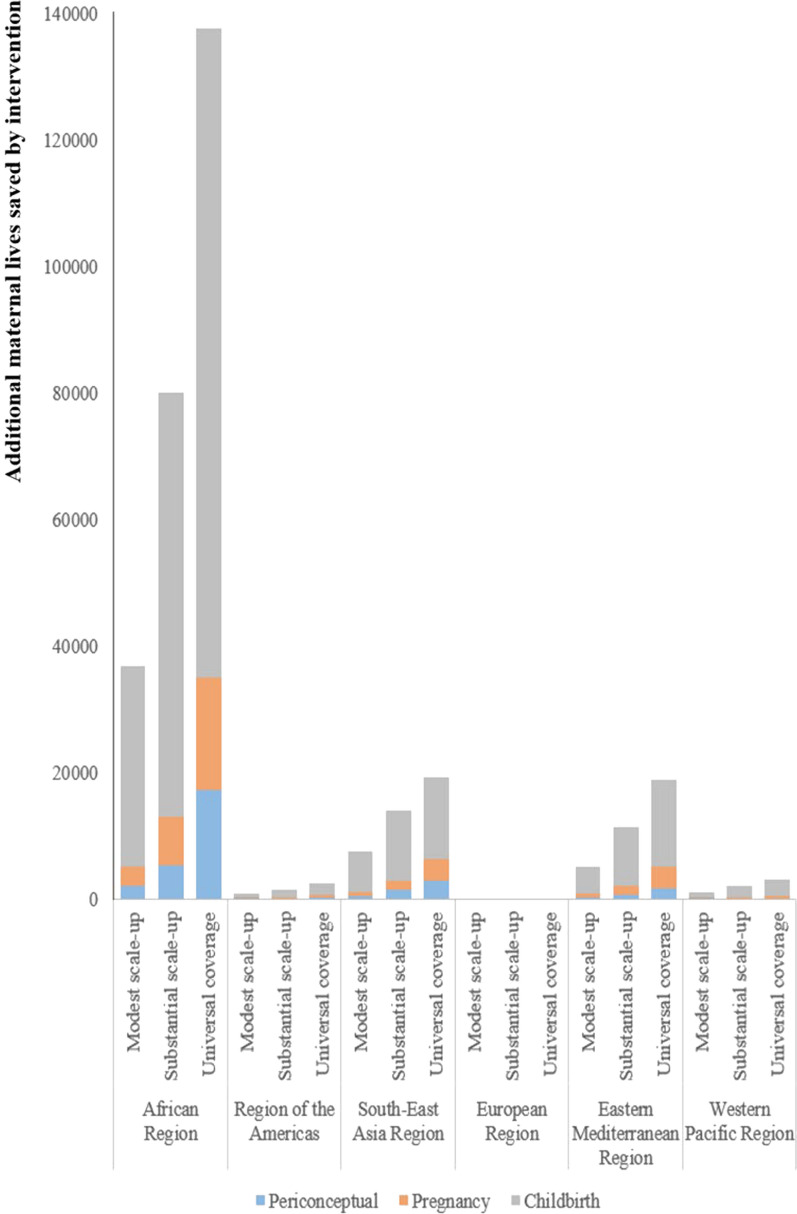
Additional maternal lives saved by intervention under three scenarios among 26 LMICs in 2030. *Notes*: Modest scale-up (Scenario 1): We assumed that coverage of every health intervention increased by 2% per year to a maximum of 100%. Substantial scale-up (Scenario 2): we assumed that coverage of every health intervention increased by 5% per year up to a maximum of 100%. Universal coverage (Scenario 3): we assumed that coverage of every health intervention would reach 95% by 2030

Additional maternal lives saved by scaling up the coverage of health interventions were mainly clustered in the African Region, the Southeast Asia Region, and the Eastern Mediterranean Region. Compared with periconceptual and pregnancy interventions, scaling up childbirth interventions could save more additional maternal lives (Fig. 2). In the 126 LMICs, additional maternal lives saved by scaling up the coverage of health interventions were 51,983, 109,618 and 181,872, respectively under scenarios 1–3. For LMICs in the African Region, additional maternal lives saved by scaling up the coverage of health interventions were 36,866, 80,164 and 137,650, respectively under scenarios 1–3. For LMICs in the Region of the Americas, additional maternal lives saved by scaling up the coverage of health interventions were 893, 1659, and 2535, respectively, under scenarios 1–3. For LMICs in the Southeast Asia Region, additional maternal lives saved by scaling up the coverage of health interventions were 7677, 14,089, and 19,262, respectively, under scenarios 1–3. For LMICs in the European Region, additional maternal lives saved by scaling up the coverage of health interventions were 73, 138 and 203, respectively under scenario 1–3. For LMICs in the Eastern Mediterranean Region, additional maternal lives saved by scaling up the coverage of health interventions were 5263, 11,418 and 19,043, respectively under scenarios 1–3. For LMICs in the Western Pacific Region, additional maternal lives saved by scaling up the coverage of health interventions were 1211, 2150 and 3179, respectively under scenario 1–3. Compared with other included interventions, uterotonics for postpartum hemorrhage, assisted vaginal delivery and cesarean delivery played more important roles in reducing maternal mortality. For the 126 LMICs, additional maternal lives saved by scaling up the coverage of uterotonics for postpartum hemorrhage were 9297, 18,295 and 19,191, respectively under scenario 1–3. Additional maternal lives saved by scaling up the coverage of assisted vaginal delivery were 3918, 9495 and 21,843, respectively under scenario 1–3. Additional maternal lives saved by scaling up the coverage of cesarean delivery were 8101, 14,722 and 18,491, respectively under scenario 1–3 (Table S6).

## Discussion

This modelling study was the first global maternal mortality study focusing on the 126 LMICs across the six WHO regions. Nearly 70% of LMICs have increased or stalled on the reduction of MMR since 2015. The leading causes of maternal deaths in the 126 LMICs were hypertensive disorders and postpartum hemorrhage. Even under a substantial scale-up of intervention coverage or scaling up to universal coverage of interventions by 2030, it would be a formidable challenge for the 126 LMICs to reach the SDG Target 3.1 on time. Additional maternal lives saved by scaling up the coverage of health interventions were mainly clustered in the African Region, the Southeast Asia Region, and the Eastern Mediterranean Region. Compared with other included interventions, uterotonics for postpartum hemorrhage, assisted vaginal delivery, and cesarean delivery deserved more attention.

There is an ongoing and urgent need for maternal health and survival to remain high within the global health and development agenda. There are both national and international estimates of MMR for each country, but different data collection and statistical methods are used, resulting in differences in MMR data. This study used MMR estimates from WHO, UNICEF, UNFPA, World Bank Group, and UNDESA/Population Division, which were widely recognized in the world. The 2020 average MMR of the included 126 LMICs was 200 maternal deaths per 100,000 live births, and significant inequities persist between regions. Regional MMRs in 2020 ranged from 17 for LMICs in the European Region to 398 for LMICs in the African Region. The MMR of each region is a direct reflection of the economic development level and a crucial indicator for assessing the health status in this region. 69.85% (88/126) of LMICs have increased or stalled on the reduction of MMR since 2015. While there has been a downward trend of MMR since 2015 among LMICs in the Africa Region, the MMR level is still far from the SDG Target 3.1. Due to the direct or indirect impact of the COVID-19, the impact of health governance, climate change and other complex social or economic factors, LMICs in another five regions have all increased or stalled on the reduction of MMR since 2015 [14]. Prioritizing, accelerated, and sustained actions are urgently required to address the stagnation of global MMR. Previous studies suggested that, except for other direct causes and indirect causes, postpartum hemorrhage was the leading cause of maternal death [9]. At the same time, our study found that hypertensive disorders accounted for the highest proportion of maternal deaths, followed by postpartum hemorrhage. Postpartum hemorrhage mainly existed in the African Region, the Southeast Asia Region and the Western Pacific Region where medical resources were scarce [15]. Hypertensive diseases were mainly found in the African Region, the Eastern Mediterranean Region, and the Region of the Americas, where the accessibility and quality of maternity service needed to be improved [16]. A study has also shown that hypertensive diseases were a leading cause of adverse maternal and perinatal outcomes [17]. It was noteworthy that this study uncovered the transition in the proportion of causes of death, particularly highlighting the importance of hypertensive disorders.

Reducing preventable maternal deaths needs long-term planning and multifaceted development [18]. Multiple determinants could affect maternal mortality, including health system failures, social determinants, harmful gender inequalities, climate, humanitarian crises and so on. Expanding the coverage of a continuum of maternity care including prenatal, intrapartum and postnatal care was the most essential element to reduce preventable maternal deaths [19]. Health interventions included in this modelling study were aligned with the reproductive, maternal, newborn, and child health (RMNCH) services mentioned in the universal health coverage (UHC) target. Increasing health intervention coverage was a joint initiative toward realizing both the SDGs and UHC. A review of 49 LMICs among six regions in 2022 indicated that effective coverage metrics took both crude coverage and quality of interventions into account [20], for example, the coverage of cesarean section on maternal request was not effective coverage of cesarean section. The effective coverage of maternal health interventions in LMICs ranged from 7 to 94%, needing to be expanded to reach the UHC. Increasing the coverage of maternal health interventions also contributed to achieving SDG Target 5, gender equality. When scaling up the coverage of maternal health interventions, mobile health technologies could be used to exchange experiences and share health resources better. At the same time, standardized data collection on maternal health interventions could be implemented in LMICs around the world, facilitating comparative analysis and resource sharing within and across regions [21].

We found that health investment in uterotonics for postpartum hemorrhage, assisted vaginal delivery and cesarean delivery could be increased and given priority attention for LMICs. Uterotonics is one of the significant preventions for postpartum hemorrhage recommended by WHO [22]. Cesarean delivery serves as a life-saving intervention for numerous emergency obstetricians, such as obstructed labor [23]. Although uterotonics for postpartum hemorrhage and cesarean section were nearly universal coverage, the effective coverage of correct use of uterotonics and necessary cesarean section still needed to be scaled up [24]. Assisted vaginal delivery plays a crucial role in averting maternal mortality and has the pediatric and maternal benefits of a vaginal birth that cesarean deliveries do not. However, many LMICs exhibited low use of assisted vaginal delivery [25].

This study estimated that with a modest, substantial, or universal scale-up, the 2030 average MMR of the 126 LMICs would be 172.05, 139.84 or 98.59, not meeting the SDG Target 3.1. Additional maternal lives saved by scaling up the coverage of health interventions were mainly clustered in the African Region, the Southeast Asia Region, and the Eastern Mediterranean Region. To cope with the stagnation of MMR and achieve the SDG Target 3.1 on time, every region needed extraordinary efforts.

For LMICs in the African Region, even under a substantial or universal scale-up, the 2030 average MMR would be 277.84 or 190.58, still far from achieving the SDG target 3.1. With low baseline maternal health intervention coverage, the African Region could substantially improve the coverage, availability, utilization and quality of health services in maternal health interventions.Additionally, the African Region could strengthen cooperation in health care and bring in specialized personnel [26].

For Southeast Asia Region and the Eastern Mediterranean Region, some low-income and lower-middle-income countries with high levels of MMR have been devastated by man-made conflicts, resulting in tremendous and profound effects on the health and well-being of the population, especially for vulnerable groups such as mothers and children [27]. Under scaling up to universal coverage, the average MMR of LMICs in the Southeast Asia Region would be 60.20. Under a modest, substantial, or universal scale-up, the average MMR of lower-middle-income countries in the Eastern Mediterranean Region would be 64.55, 54.12 or 43.38. For LMICs in the Southeast Asia Region and the Eastern Mediterranean Region, although it’s challenging to scale up the coverage of maternal health interventions substantially or reach universal coverage, for meeting the SDG Target 3.1, coordinated and best efforts are needed to scale up the coverage of a continuum of care in maternal health interventions.

For LMICs in the Region of the Americas and Western Pacific Region, with a substantial or universal scale-up, the average MMR of LMICs in the Region of the Americas would be 63.09 or 49.83 while the average MMR of LMICs in Western Pacific Region would be 58.35 or 46.15, both meeting SDG target 3.1. A modest scale-up of maternal health intervention coverage is feasible for the Region of the Americas and the Western Pacific Region. According to studies in these two regions, for reducing maternal deaths and reaching the SDG target 3.1 on time, on the basis of achieving a modest scale-up, paramount importance could be placed on increasing the quality of obstetric interventions, improving the efficiency and fairness of the government health funding, and strengthening the health system [28, 29]. This study further estimated that by increasing the coverage of childbirth interventions by 5% per year or scaling up the coverage of childbirth interventions to 95%, the average MMR of LMICs in the Region of the Americas would be 67.88 or 59.42, and the average MMR of LMICs in the Western Pacific Region would be 63.32 or 53.08, achieving the SDG Target 3.1 (Table S6). For LMICs in the European Region they could sustain and improve system capabilities and sector collaborations for mortality reduction. LMICs in the six regions could strengthen global collaborations, promote the exchange of good health governance experience and medical resources to reduce maternal mortality and reach the SDG Target 3.1 together.

This modelling study has several limitations. Firstly, using the APC of 2015–2020 to predict the MMR trend of 2020–2030 may have some deviations from the actual situation, but the joinpoint regression model has good stability and reliability. Secondly, there was a discrepancy between modelling study estimating findings and the real world. However, the default assumptions in LiST model about baseline health status, population size, and intervention effectiveness were best available and comprehensive. In that the modelling results were indicative and directional [6–8]. Thirdly, because the LiST model can only adjust for health intervention coverage, this modelling study only estimated the impacts of health intervention coverage on reducing maternal mortality. Future research could incorporate the quality and accessibility of health interventions, as well as other political and cultural factors related to maternal mortality into the model. Fourthly, due to lags in data reporting, we used MMR data in 2020, data on the causes of maternal deaths in 2021 instead of the data in 2024 as the baseline data, but the data was comprehensive and latest, and included the potential impact of the COVID-19.

## Conclusions

Nearly 70% of LMICs have increased or stalled on the reduction of MMR globally since 2015. Except for other direct and indirect causes, hypertensive disorders and postpartum hemorrhage were the leading causes of maternal deaths in LMICs. This study suggested that even under a substantial or universal scale-up of intervention coverage by 2030, the 2030 average MMR of the 126 LMICs could not be reduced to less than 70, in other words, it is challenging for LMICs to reach the SDG Target 3.1 on time. Accelerated actions are urgently needed to scale up the effective coverage of maternal health interventions, optimize the allocation of health resources, promote health equality, take more decisive national, regional and global actions. Our study findings provided scientific evidence for making national-, regional-, and global-level health policies for LMICs to cope with the stagnation of MMR and end preventable maternal deaths earlier. Future researches could apply updated data and optimize the model to further verify and expand the results of this study.

## Supplementary Information

Supplementary Material 1.

## Data Availability

The data supporting this study's findings are available from WHO and GBD 2021.
