# Supplementary material for: Impact of health intervention coverage on reducing maternal mortality in 126 low- and middle-income countries: a Lives Saved Tool modelling study
Source: Glob Health Res Policy. 2025 Apr 2;10:15. doi: 10.1186/s41256-025-00414-0 (PMC11963500; doi:10.1186/s41256-025-00414-0)
Supplement: Supplementary file 1 — Supplementary Material 1. [file 41256_2025_414_MOESM1_ESM.docx]

**Table S1 LMICs included in the study**

| **Region** | **Country** |
| --- | --- |
| WHO African Region | Algeria, Angola, Benin, Botswana, Burkina Faso, Burundi, Cabo Verde, Cameroon, Central African Republic, Chad, Comoros, Congo, Cote d'Ivoire, Democratic Republic of the Congo, Equatorial Guinea, Eritrea, Ethiopia, Gabon, Gambia, Ghana, Guinea, Guinea-Bissau, Kenya, Lesotho, Liberia, Madagascar, Malawi, Mali, Mauritania, Mauritius, Mozambique, Namibia, Niger, Nigeria, Rwanda, Sao Tome and Principe, Senegal, Sierra Leone, South Africa, South Sudan, Togo, Uganda, United Republic of Tanzania, Zambia, Zimbabwe |
| WHO Region of the Americas | Argentina, Belize, Bolivia, Brazil, Colombia, Costa Rica, Cuba, Dominican Republic, Ecuador, El Salvador, Grenada, Guatemala, Haiti, Honduras, Jamaica, Mexico, Nicaragua, Paraguay, Peru, Saint Lucia, Saint Vincent and the Grenadines, Suriname |
| WHO Southeast Asia Region | Bangladesh, Bhutan, Dem. People's Republic of Korea, India, Indonesia, Maldives, Myanmar, Nepal, Sri Lanka, Thailand, Timor-Leste |
| WHO European Region | Albania, Armenia, Azerbaijan, Belarus, Bosnia and Herzegovina, Georgia, Kazakhstan, Kyrgyzstan, Montenegro, Republic of Moldova, Republic of North Macedonia, Serbia, Tajikistan, Turkey, Turkmenistan, Ukraine, Uzbekistan |
| WHO Eastern Mediterranean Region | Afghanistan, Djibouti, Egypt, Iran(Islamic Republic of), Iraq, Jordan, Lebanon, Libyan Arab Jamahiriya, Morocco, Occupied Palestinian Territory, Pakistan, Somalia, Sudan, Syrian Arab Republic, Tunisia, Yemen |
| WHO Western Pacific Region | Cambodia, China, Fiji, Kiribati, Lao Peope's Democratic Republic, Malaysia, Micronesia (Fed. States of), Mongolia, Papua New Guinea, Philippines, Samoa, Solomon Islands, Tonga, Vanuatu, Viet Nam |

**Table S2 Default efficacy and affected factor in the LiST (Lives Saved Tool)**

| **Causes of maternal death** | **Intervention** | **Efficacy** | **Affected fraction** |
| --- | --- | --- | --- |
| **Intrapartum hemorrhage** |  |  |  |
|  | Blood transfusion | 0.50 | 1.00 |
| **Postpartum hemorrhage** |  |  |  |
|  | Uterotonics for postpartum hemorrhage | 0.78 | 1.00 |
|  | Manual removal of placenta | 0.30 | 1.00 |
|  | Removal of retained products of conception | 0.30 | 1.00 |
|  | Blood transfusion | 0.50 | 1.00 |
| **Hypertensive disorders** |  |  |  |
|  | Hypertensive disorder case management | 0.50 | 1.00 |
|  | MgSO_4_ for eclampsia | 0.59 | 1.00 |
|  | Cesarean delivery | 0.45 | 1.00 |
| **Sepsis** |  |  |  |
|  | Clean birth environment | 0.60 | 1.00 |
|  | Antibiotics for preterm or prolonged PROM | 0.80 | 0.33 |
|  | Antibiotics for maternal sepsis | 0.80 | 1.00 |
|  | Blood transfusion | 0.25 | 1.00 |
| **Abortion** |  |  |  |
|  | Safe abortion services | 0.95 | 0.82 |
| **Other direct causes** |  |  |  |
|  | Assisted vaginal delivery | 0.39 | 1.00 |
|  | Cesarean delivery | 0.90 | 0.50 |
| **Indirect causes** |  |  |  |
|  | TT - Tetanus toxoid vaccination | 0.98 | 0.01 |

**Table S3** **MMR and APC, AAPC of MMR in 2000~2020 among 126 LMICs**

|  | **MMR** | | | | | **APC** | | | **AAPC** |
| --- | --- | --- | --- | --- | --- | --- | --- | --- | --- |
|  | 2000 | 2005 | 2010 | 2015 | 2020 | 2000~2010 | 2010~2015 | 2015~2020 |  |
| **Worldwide** | **354** | **296** | **262** | **223** | **200** | **-3.74** | **-3.26** | **-1.47** | **-2.97** |
| Afghanistan | 1346 | 1103 | 899 | 776 | 620 | -4.09 | -3.52 | -4.08 | -3.80 |
| Albania | 14 | 11 | 9 | 7 | 8 | -4.62 | -4.31 | 2.37 | -2.76 |
| Algeria | 159 | 144 | 112 | 89 | 78 | -2.01 | -4.72 | -2.51 | -3.50 |
| Angola | 860 | 550 | 367 | 274 | 222 | -8.94 | -6.72 | -3.73 | -6.55 |
| Argentina | 72 | 63 | 55 | 39 | 45 | -1.59 | -4.99 | 2.48 | -2.32 |
| Armenia | 50 | 38 | 33 | 25 | 27 | -4.92 | -4.02 | 0.94 | -3.03 |
| Azerbaijan | 56 | 44 | 33 | 29 | 41 | -5.00 | -4.35 | 8.10 | -1.55 |
| Bangladesh | 441 | 376 | 301 | 212 | 123 | -2.73 | -5.52 | -10.78 | -6.18 |
| Belarus | 24 | 11 | 3 | 1 | 1 | -14.78 | -21.68 | 1.31 | -14.69 |
| Belize | 86 | 72 | 33 | 49 | 130 | -6.95 | -4.08 | 26.86 | 2.09 |
| Benin | 469 | 509 | 598 | 591 | 523 | 2.18 | 1.61 | -3.13 | 0.55 |
| Bhutan | 305 | 186 | 117 | 74 | 60 | -9.43 | -8.81 | -4.09 | -7.81 |
| Bolivia | 284 | 230 | 184 | 165 | 161 | -4.38 | -3.43 | 0.10 | -2.80 |
| Bosnia and Herzegovina | 16 | 11 | 8 | 7 | 6 | -7.89 | -4.34 | -2.50 | -4.79 |
| Botswana | 182 | 201 | 156 | 184 | 186 | -0.15 | -0.37 | 1.34 | 0.11 |
| Brazil | 68 | 70 | 64 | 62 | 72 | 0.32 | -1.17 | 3.23 | 0.29 |
| Burkina Faso | 506 | 417 | 357 | 295 | 264 | -3.72 | -3.34 | -2.39 | -3.20 |
| Burundi | 874 | 713 | 608 | 514 | 494 | -3.96 | -3.22 | -0.83 | -2.81 |
| Cabo Verde | 125 | 77 | 54 | 47 | 42 | -9.89 | -4.95 | -1.23 | -5.31 |
| Cambodia | 606 | 382 | 276 | 209 | 218 | -8.92 | -5.91 | 1.10 | -4.98 |
| Cameroon | 651 | 573 | 527 | 447 | 438 | -2.30 | -2.40 | -0.75 | -1.96 |
| Central African Republic | 1315 | 1158 | 1052 | 909 | 835 | -2.39 | -2.35 | -1.89 | -2.25 |
| Chad | 1366 | 1241 | 1303 | 1125 | 1063 | -1.41 | -0.73 | -2.12 | -1.25 |
| China | 58 | 46 | 33 | 26 | 23 | -4.83 | -5.56 | -2.07 | -4.52 |
| Colombia | 93 | 82 | 71 | 70 | 75 | -2.66 | -1.82 | 2.09 | -1.07 |
| Comoros | 456 | 360 | 316 | 261 | 217 | -4.44 | -3.13 | -3.86 | -3.64 |
| Congo | 660 | 488 | 389 | 360 | 282 | -6.24 | -3.16 | -4.05 | -4.16 |
| Costa Rica | 39 | 31 | 27 | 20 | 22 | -4.13 | -4.04 | 1.01 | -2.82 |
| Cote d'Ivoire | 473 | 540 | 604 | 530 | 480 | 3.23 | 0.17 | -3.17 | 0.07 |
| Cuba | 47 | 43 | 41 | 39 | 39 | -1.75 | -0.97 | -0.01 | -0.93 |
| Dem. People's Republic of Korea | 186 | 122 | 130 | 108 | 107 | -7.47 | -1.03 | -1.21 | -2.73 |
| Democratic Republic of the Congo | 668 | 635 | 601 | 578 | 547 | -1.05 | -0.96 | -1.00 | -0.99 |
| Djibouti | 512 | 368 | 274 | 244 | 234 | -6.89 | -4.11 | -0.14 | -3.84 |
| Dominican Republic | 79 | 87 | 92 | 99 | 107 | 1.90 | 1.28 | 1.65 | 1.53 |
| Ecuador | 120 | 92 | 76 | 66 | 66 | -5.38 | -3.25 | 0.18 | -2.95 |
| Egypt | 79 | 58 | 38 | 24 | 17 | -5.89 | -8.41 | -6.84 | -7.39 |
| El Salvador | 49 | 35 | 36 | 44 | 43 | -6.78 | 1.98 | 0.49 | -0.65 |
| Equatorial Guinea | 427 | 258 | 211 | 201 | 212 | -9.95 | -2.60 | 1.76 | -3.44 |
| Eritrea | 735 | 546 | 480 | 399 | 322 | -5.66 | -3.01 | -4.46 | -4.04 |
| Ethiopia | 953 | 880 | 635 | 399 | 267 | -1.32 | -7.34 | -8.49 | -6.16 |
| Fiji | 49 | 45 | 42 | 39 | 38 | -1.68 | -1.41 | -0.56 | -1.26 |
| Gabon | 249 | 239 | 193 | 212 | 227 | -1.55 | -1.58 | 2.94 | -0.46 |
| Gambia | 778 | 718 | 620 | 535 | 458 | -1.59 | -2.90 | -3.06 | -2.61 |
| Georgia | 53 | 45 | 41 | 30 | 28 | -2.50 | -4.03 | -1.99 | -3.14 |
| Ghana | 499 | 390 | 337 | 286 | 263 | -4.75 | -3.05 | -1.74 | -3.15 |
| Grenada | 42 | 32 | 27 | 23 | 21 | -5.32 | -3.25 | -1.77 | -3.41 |
| Guatemala | 152 | 149 | 123 | 107 | 96 | -0.55 | -3.29 | -1.93 | -2.27 |
| Guinea | 971 | 846 | 741 | 649 | 553 | -2.72 | -2.62 | -3.15 | -2.78 |
| Guinea-Bissau | 1300 | 977 | 795 | 713 | 725 | -5.86 | -3.15 | 0.75 | -2.88 |
| Haiti | 400 | 363 | 403 | 391 | 350 | -1.70 | 0.82 | -2.55 | -0.67 |
| Honduras | 82 | 75 | 73 | 67 | 72 | -1.43 | -1.13 | 1.11 | -0.65 |
| India | 384 | 286 | 179 | 128 | 103 | -6.35 | -7.46 | -4.17 | -6.37 |
| Indonesia | 299 | 276 | 219 | 194 | 173 | -1.80 | -3.65 | -1.67 | -2.70 |
| Iran (Islamic Republic of) | 44 | 30 | 32 | 20 | 22 | -6.21 | -2.80 | -1.76 | -3.41 |
| Iraq | 117 | 116 | 115 | 102 | 76 | 0.23 | -1.25 | -6.14 | -2.13 |
| Jamaica | 83 | 86 | 88 | 97 | 99 | 0.49 | 1.19 | 0.68 | 0.89 |
| Jordan | 64 | 58 | 47 | 46 | 41 | -2.39 | -2.55 | -1.31 | -2.20 |
| Kazakhstan | 56 | 36 | 20 | 13 | 13 | -9.06 | -9.81 | 0.93 | -7.04 |
| Kenya | 564 | 503 | 476 | 483 | 530 | -2.43 | -0.50 | 2.25 | -0.31 |
| Kiribati | 116 | 118 | 131 | 121 | 76 | 0.82 | 0.41 | -9.59 | -2.09 |
| Kyrgyzstan | 87 | 83 | 72 | 61 | 50 | -0.86 | -3.02 | -3.99 | -2.73 |
| Lao Peope's Democratic Republic | 579 | 442 | 284 | 184 | 126 | -5.28 | -8.40 | -7.26 | -7.34 |
| Lebanon | 32 | 25 | 18 | 19 | 21 | -6.79 | -2.05 | 2.79 | -2.08 |
| Lesotho | 545 | 563 | 1040 | 728 | 566 | 2.66 | 4.27 | -9.73 | 0.19 |
| Liberia | 777 | 676 | 634 | 686 | 652 | -3.20 | 0.07 | -0.40 | -0.87 |
| Libyan Arab Jamahiriya | 57 | 50 | 57 | 71 | 72 | -2.82 | 3.51 | 0.63 | 1.17 |
| Madagascar | 658 | 568 | 497 | 482 | 392 | -3.20 | -1.65 | -3.71 | -2.56 |
| Malawi | 573 | 347 | 513 | 445 | 381 | -8.18 | 2.88 | -5.16 | -2.02 |
| Malaysia | 40 | 32 | 25 | 22 | 21 | -4.49 | -4.03 | -0.06 | -3.17 |
| Maldives | 114 | 79 | 60 | 57 | 57 | -7.79 | -3.24 | 0.84 | -3.41 |
| Mali | 742 | 560 | 547 | 494 | 440 | -5.26 | -1.19 | -2.63 | -2.58 |
| Mauritania | 684 | 652 | 586 | 510 | 464 | -0.85 | -2.43 | -1.98 | -1.92 |
| Mauritius | 52 | 48 | 55 | 57 | 84 | -1.22 | 1.75 | 7.63 | 2.43 |
| Mexico | 57 | 53 | 51 | 52 | 59 | -1.61 | -0.24 | 2.83 | 0.17 |
| Micronesia (Fed. States of) | 60 | 57 | 46 | 64 | 74 | -2.53 | 0.87 | 5.14 | 1.05 |
| Mongolia | 158 | 94 | 65 | 47 | 39 | -9.96 | -6.77 | -3.40 | -6.76 |
| Montenegro | 11 | 10 | 7 | 6 | 6 | -2.41 | -5.15 | 0.90 | -2.99 |
| Morocco | 244 | 194 | 134 | 97 | 72 | -4.63 | -6.75 | -5.53 | -5.92 |
| Mozambique | 532 | 404 | 322 | 226 | 127 | -4.99 | -5.61 | -11.30 | -6.91 |
| Myanmar | 371 | 321 | 293 | 243 | 179 | -2.59 | -2.65 | -6.37 | -3.58 |
| Namibia | 450 | 419 | 482 | 299 | 215 | -0.10 | -2.36 | -9.43 | -3.63 |
| Nepal | 504 | 380 | 349 | 252 | 174 | -5.00 | -3.39 | -8.83 | -5.18 |
| Nicaragua | 169 | 158 | 98 | 79 | 78 | -2.37 | -6.56 | 0.50 | -3.79 |
| Niger | 867 | 768 | 594 | 491 | 441 | -2.54 | -4.48 | -1.77 | -3.32 |
| Nigeria | 1148 | 1073 | 1123 | 1113 | 1047 | -1.16 | 0.40 | -1.47 | -0.46 |
| Occupied Palestinian Territory | 62 | 54 | 43 | 26 | 20 | -0.48 | -7.89 | -5.56 | -5.50 |
| Pakistan | 387 | 301 | 230 | 187 | 154 | -5.19 | -4.60 | -3.62 | -4.50 |
| Papua New Guinea | 312 | 314 | 289 | 208 | 192 | 1.02 | -4.05 | -2.43 | -2.40 |
| Paraguay | 148 | 127 | 100 | 80 | 71 | -3.10 | -4.48 | -2.34 | -3.61 |
| Peru | 113 | 97 | 76 | 65 | 69 | -3.29 | -4.01 | 1.69 | -2.44 |
| Philippines | 129 | 122 | 105 | 88 | 78 | -1.04 | -3.19 | -2.50 | -2.48 |
| Republic of Moldova | 49 | 30 | 18 | 17 | 12 | -10.75 | -5.80 | -4.69 | -6.79 |
| Republic of North Macedonia | 12 | 9 | 6 | 5 | 3 | -5.82 | -5.97 | -9.00 | -6.70 |
| Rwanda | 1007 | 533 | 386 | 312 | 259 | -12.20 | -5.31 | -3.19 | -6.56 |
| Saint Lucia | 87 | 82 | 73 | 80 | 73 | -1.76 | -0.44 | -0.85 | -0.87 |
| Saint Vincent and the Grenadines | 76 | 54 | 45 | 36 | 62 | -6.49 | -3.95 | 11.28 | -1.01 |
| Samoa | 75 | 62 | 62 | 58 | 59 | -3.55 | -0.62 | 0.07 | -1.19 |
| Sao Tome and Principe | 179 | 160 | 160 | 139 | 146 | -1.89 | -1.27 | 0.38 | -1.01 |
| Senegal | 638 | 519 | 450 | 321 | 261 | -3.54 | -4.51 | -4.91 | -4.37 |
| Serbia | 18 | 16 | 14 | 13 | 10 | -2.57 | -2.07 | -4.85 | -2.90 |
| Sierra Leone | 1682 | 1327 | 837 | 588 | 443 | -5.04 | -7.80 | -5.14 | -6.45 |
| Solomon Islands | 150 | 153 | 147 | 141 | 122 | 0.40 | -0.81 | -2.86 | -1.03 |
| Somalia | 1097 | 1080 | 963 | 761 | 621 | 0.04 | -3.39 | -4.42 | -2.80 |
| South Africa | 173 | 221 | 219 | 141 | 127 | 5.96 | -3.91 | -3.92 | -1.53 |
| South Sudan | 1687 | 1332 | 1060 | 1225 | 1223 | -5.34 | -1.48 | 2.05 | -1.60 |
| Sri Lanka | 61 | 44 | 37 | 30 | 29 | -6.24 | -3.74 | -0.80 | -3.65 |
| Sudan | 642 | 525 | 383 | 298 | 270 | -4.24 | -5.51 | -1.64 | -4.24 |
| Suriname | 278 | 190 | 138 | 125 | 96 | -8.81 | -3.75 | -4.31 | -5.18 |
| Syrian Arab Republic | 34 | 23 | 21 | 30 | 30 | -8.46 | 1.23 | 3.96 | -0.62 |
| Tajikistan | 68 | 44 | 32 | 20 | 17 | -8.01 | -7.44 | -3.84 | -6.70 |
| Thailand | 48 | 40 | 35 | 30 | 29 | -3.49 | -2.86 | -0.72 | -2.49 |
| Timor-Leste | 750 | 584 | 376 | 285 | 204 | -5.47 | -6.93 | -5.87 | -6.30 |
| Togo | 479 | 494 | 530 | 441 | 399 | 1.36 | -0.94 | -3.06 | -0.91 |
| Tonga | 94 | 95 | 93 | 86 | 126 | 0.37 | -0.96 | 7.71 | 1.48 |
| Tunisia | 62 | 56 | 44 | 40 | 37 | -2.76 | -3.43 | -0.54 | -2.55 |
| Turkey | 32 | 25 | 22 | 19 | 17 | -4.73 | -2.73 | -2.24 | -3.11 |
| Turkmenistan | 26 | 17 | 9 | 6 | 5 | -8.53 | -10.38 | -2.12 | -7.91 |
| Uganda | 461 | 435 | 372 | 319 | 284 | -1.16 | -3.06 | -2.28 | -2.39 |
| Ukraine | 36 | 21 | 17 | 11 | 17 | -9.63 | -6.07 | 7.95 | -3.68 |
| United Republic of Tanzania | 760 | 559 | 486 | 330 | 238 | -5.43 | -4.65 | -7.80 | -5.64 |
| Uzbekistan | 43 | 45 | 38 | 31 | 30 | 1.04 | -3.67 | -0.76 | -1.78 |
| Vanuatu | 109 | 98 | 93 | 92 | 94 | -2.21 | -0.65 | 0.59 | -0.74 |
| Viet Nam | 88 | 66 | 60 | 52 | 46 | -5.44 | -2.35 | -2.60 | -3.19 |
| Yemen | 275 | 196 | 157 | 164 | 183 | -7.20 | -2.16 | 3.76 | -2.02 |
| Zambia | 419 | 309 | 268 | 166 | 135 | -5.22 | -5.60 | -5.61 | -5.51 |
| Zimbabwe | 388 | 533 | 618 | 408 | 357 | 8.19 | -1.93 | -5.48 | -0.42 |

Notes：Maternal mortality ratio (MMR) defined as maternal deaths per 100,000 live births for women of reproductive age (15~49 years). APC was annual percentage change and AAPC was average annual percentage change calculated by joinpoint regression.

**Table S4 Percent of maternal deaths in 2021 by cause among six WHO regions**

|  | **Antepartum hemorrhage (%)** | **Intrapartum hemorrhage (%)** | **Postpartum hemorrhage (%)** | **Hypertensive disorders (%)** | **Sepsis (%)** | **Abortion (%)** | **Other direct causes (%)** | **Indirect causes (%)** |
| --- | --- | --- | --- | --- | --- | --- | --- | --- |
| **Worldwide** | 4.40 | 0.55 | 13.57 | 15.78 | 5.93 | 6.72 | 39.20 | 13.85 |
| **African Region** | 4.29 | 0.54 | 13.21 | 16.86 | 8.11 | 8.35 | 37.08 | 11.56 |
| **Region of the Americas** | 3.50 | 0.44 | 10.78 | 19.00 | 5.10 | 5.99 | 37.64 | 17.55 |
| **South-East Asia Region** | 6.48 | 0.81 | 19.97 | 15.14 | 3.51 | 6.28 | 32.17 | 15.64 |
| **European Region** | 3.75 | 0.47 | 11.57 | 13.20 | 4.26 | 4.41 | 44.88 | 17.46 |
| **Eastern Mediterranean Region** | 4.28 | 0.54 | 13.18 | 15.58 | 5.93 | 6.12 | 42.22 | 12.15 |
| **Western Pacific Region** | 5.44 | 0.68 | 16.74 | 11.41 | 4.25 | 6.42 | 43.36 | 11.70 |

**Table S5 Estimated relative reductions of** **MMR by 2030 in four scenarios among 126 LMICs**

|  | **Scenario 0 (No scale-up)** | **Scenario 1 (Modest scale-up)** | | **Scenario 2 (Substantial scale-up)** | | **Scenario 3 (Universal coverage)** | |
| --- | --- | --- | --- | --- | --- | --- | --- |
|  | MMR  (per 100,000 live births) | MMR  (per 100,000 live births) | Reduction  (%) | MMR  (per 100,000 live births) | Reduction  (%) | MMR  (per 100,000 live births) | Reduction  (%) |
| **Worldwide** | **200** | **172.05** | **13.81** | **139.84** | **27.39** | **98.59** | **42.53** |
| Afghanistan | 620 | 519.74 | 16.17 | 388.82 | 37.29 | 236.42 | 61.87 |
| Albania | 8 | 7.10 | 11.25 | 6.56 | 18.00 | 6.78 | 15.25 |
| Algeria | 78 | 70.96 | 9.03 | 65.75 | 15.71 | 62.34 | 20.08 |
| Angola | 222 | 191.69 | 13.65 | 148.32 | 33.19 | 81.80 | 63.15 |
| Argentina | 45 | 40.29 | 10.47 | 36.37 | 19.18 | 30.20 | 32.89 |
| Armenia | 27 | 25.71 | 4.78 | 25.08 | 7.11 | 24.91 | 7.74 |
| Azerbaijan | 41 | 37.46 | 8.63 | 32.85 | 19.88 | 26.27 | 35.93 |
| Bangladesh | 123 | 107.66 | 12.47 | 88.15 | 28.33 | 66.65 | 45.81 |
| Belarus | 1 | 0.50 | 50.00 | 0.30 | 70.00 | 0.20 | 80.00 |
| Belize | 130 | 110.79 | 14.78 | 97.05 | 25.35 | 91.42 | 29.68 |
| Benin | 523 | 458.70 | 12.29 | 397.08 | 24.08 | 315.81 | 39.62 |
| Bhutan | 60 | 52.75 | 12.08 | 45.04 | 24.93 | 36.10 | 39.83 |
| Bolivia | 161 | 134.19 | 16.65 | 105.62 | 34.40 | 77.76 | 51.70 |
| Bosnia and Herzegovina | 6 | 5.00 | 16.67 | 4.39 | 26.83 | 3.88 | 35.33 |
| Botswana | 186 | 163.99 | 11.83 | 138.95 | 25.30 | 107.94 | 41.97 |
| Brazil | 72 | 63.83 | 11.35 | 58.25 | 19.10 | 51.95 | 27.85 |
| Burkina Faso | 264 | 222.81 | 15.60 | 183.37 | 30.54 | 125.66 | 52.40 |
| Burundi | 494 | 417.60 | 15.47 | 314.60 | 36.32 | 217.11 | 56.05 |
| Cabo Verde | 42 | 37.71 | 10.21 | 31.53 | 24.93 | 22.09 | 47.40 |
| Cambodia | 218 | 174.30 | 20.05 | 133.34 | 38.83 | 96.15 | 55.89 |
| Cameroon | 438 | 389.79 | 11.01 | 325.13 | 25.77 | 237.14 | 45.86 |
| Central African Republic | 835 | 723.50 | 13.35 | 559.08 | 33.04 | 276.08 | 66.94 |
| Chad | 1063 | 938.04 | 11.76 | 745.13 | 29.90 | 347.60 | 67.30 |
| China | 23 | 19.83 | 13.78 | 16.92 | 26.43 | 11.24 | 51.13 |
| Colombia | 75 | 67.42 | 10.11 | 61.91 | 17.45 | 54.87 | 26.84 |
| Comoros | 217 | 187.11 | 13.77 | 154.40 | 28.85 | 108.85 | 49.84 |
| Congo | 282 | 239.76 | 14.98 | 190.46 | 32.46 | 135.65 | 51.90 |
| Costa Rica | 22 | 19.82 | 9.91 | 18.45 | 16.14 | 18.49 | 15.95 |
| Cote d'Ivoire | 480 | 413.29 | 13.90 | 329.14 | 31.43 | 259.74 | 45.89 |
| Cuba | 39 | 36.18 | 7.23 | 33.46 | 14.21 | 30.85 | 20.90 |
| Dem. People's Republic of Korea | 107 | 91.37 | 14.61 | 75.36 | 29.57 | 48.15 | 55.00 |
| Democratic Republic of the Congo | 547 | 470.96 | 13.90 | 365.31 | 33.22 | 200.23 | 63.39 |
| Djibouti | 234 | 204.63 | 12.55 | 174.61 | 25.38 | 143.41 | 38.71 |
| Dominican Republic | 107 | 96.57 | 9.75 | 89.05 | 16.78 | 85.54 | 20.06 |
| Ecuador | 66 | 57.15 | 13.41 | 49.00 | 25.76 | 39.54 | 40.09 |
| Egypt | 17 | 14.66 | 13.76 | 12.16 | 28.47 | 9.53 | 43.94 |
| El Salvador | 43 | 38.98 | 9.35 | 35.16 | 18.23 | 29.41 | 31.60 |
| Equatorial Guinea | 212 | 188.01 | 11.32 | 154.83 | 26.97 | 113.73 | 46.35 |
| Eritrea | 322 | 275.20 | 14.53 | 216.86 | 32.65 | 113.66 | 64.70 |
| Ethiopia | 267 | 234.72 | 12.09 | 190.59 | 28.62 | 130.59 | 51.09 |
| Fiji | 38 | 31.89 | 16.08 | 27.63 | 27.29 | 23.45 | 38.29 |
| Gabon | 227 | 195.79 | 13.75 | 164.20 | 27.67 | 145.92 | 35.72 |
| Gambia | 458 | 399.49 | 12.78 | 318.01 | 30.57 | 217.82 | 52.44 |
| Georgia | 28 | 25.16 | 10.14 | 22.56 | 19.43 | 19.31 | 31.04 |
| Ghana | 263 | 232.15 | 11.73 | 200.66 | 23.70 | 181.64 | 30.94 |
| Grenada | 21 | 18.48 | 12.00 | 15.04 | 28.38 | 7.43 | 64.62 |
| Guatemala | 96 | 82.51 | 14.05 | 66.62 | 30.60 | 45.61 | 52.49 |
| Guinea | 553 | 488.20 | 11.72 | 388.23 | 29.80 | 230.26 | 58.36 |
| Guinea-Bissau | 725 | 630.58 | 13.02 | 505.73 | 30.24 | 297.59 | 58.95 |
| Haiti | 350 | 287.27 | 17.92 | 203.27 | 41.92 | 96.71 | 72.37 |
| Honduras | 72 | 60.89 | 15.43 | 50.52 | 29.83 | 41.15 | 42.85 |
| India | 103 | 81.91 | 20.48 | 64.17 | 37.70 | 49.97 | 51.49 |
| Indonesia | 173 | 133.73 | 22.70 | 101.70 | 41.21 | 81.07 | 53.14 |
| Iran(Islamic Republic of) | 22 | 20.06 | 8.82 | 17.95 | 18.41 | 14.99 | 31.86 |
| Iraq | 76 | 65.93 | 13.25 | 56.46 | 25.71 | 51.90 | 31.71 |
| Jamaica | 99 | 86.37 | 12.76 | 76.03 | 23.20 | 65.29 | 34.05 |
| Jordan | 41 | 36.32 | 11.41 | 32.77 | 20.07 | 29.82 | 27.27 |
| Kazakhstan | 13 | 12.19 | 6.23 | 11.92 | 8.31 | 12.01 | 7.62 |
| Kenya | 530 | 432.75 | 18.35 | 358.73 | 32.32 | 322.75 | 39.10 |
| Kiribati | 76 | 57.78 | 23.97 | 42.63 | 43.91 | 36.66 | 51.76 |
| Kyrgyzstan | 50 | 42.57 | 14.86 | 35.16 | 29.68 | 30.73 | 38.54 |
| Lao Peope's Democratic Republic | 126 | 108.58 | 13.83 | 84.26 | 33.13 | 58.85 | 53.29 |
| Lebanon | 21 | 19.39 | 7.67 | 17.00 | 19.05 | 10.82 | 48.48 |
| Lesotho | 566 | 473.50 | 16.34 | 415.86 | 26.53 | 414.42 | 26.78 |
| Liberia | 652 | 586.27 | 10.08 | 498.48 | 23.55 | 386.26 | 40.76 |
| Libyan Arab Jamahiriya | 72 | 66.35 | 7.85 | 58.55 | 18.68 | 37.68 | 47.67 |
| Madagascar | 392 | 338.26 | 13.71 | 263.71 | 32.73 | 130.05 | 66.82 |
| Malawi | 381 | 324.10 | 14.93 | 271.03 | 28.86 | 217.23 | 42.98 |
| Malaysia | 21 | 18.90 | 10.00 | 16.80 | 20.00 | 13.28 | 36.76 |
| Maldives | 57 | 49.67 | 12.86 | 44.26 | 22.35 | 41.98 | 26.35 |
| Mali | 440 | 382.93 | 12.97 | 311.02 | 29.31 | 226.15 | 48.60 |
| Mauritania | 464 | 422.29 | 8.99 | 365.08 | 21.32 | 275.00 | 40.73 |
| Mauritius | 84 | 74.41 | 11.42 | 65.79 | 21.68 | 51.67 | 38.49 |
| Mexico | 59 | 52.44 | 11.12 | 47.89 | 18.83 | 42.10 | 28.64 |
| Micronesia (Fed. States of) | 74 | 65.11 | 12.01 | 54.98 | 25.70 | 35.08 | 52.59 |
| Mongolia | 39 | 33.55 | 13.97 | 30.77 | 21.10 | 31.34 | 19.64 |
| Montenegro | 6 | 5.40 | 10.00 | 4.95 | 17.50 | 4.45 | 25.83 |
| Morocco | 72 | 63.87 | 11.29 | 53.17 | 26.15 | 38.95 | 45.90 |
| Mozambique | 127 | 112.70 | 11.26 | 92.33 | 27.30 | 57.69 | 54.57 |
| Myanmar | 179 | 141.50 | 20.95 | 107.10 | 40.17 | 65.26 | 63.54 |
| Namibia | 215 | 172.18 | 19.92 | 144.16 | 32.95 | 140.61 | 34.60 |
| Nepal | 174 | 144.61 | 16.89 | 122.53 | 29.58 | 121.66 | 30.08 |
| Nicaragua | 78 | 65.15 | 16.47 | 55.00 | 29.49 | 42.26 | 45.82 |
| Niger | 441 | 390.35 | 11.49 | 310.86 | 29.51 | 159.71 | 63.78 |
| Nigeria | 1047 | 871.23 | 16.79 | 645.36 | 38.36 | 341.53 | 67.38 |
| Occupied Palestinian Territory | 20 | 18.23 | 8.85 | 17.94 | 10.30 | 18.50 | 7.50 |
| Pakistan | 154 | 126.26 | 18.01 | 95.66 | 37.88 | 70.72 | 54.08 |
| Papua New Guinea | 192 | 163.49 | 14.85 | 131.64 | 31.44 | 95.20 | 50.42 |
| Paraguay | 71 | 62.07 | 12.58 | 54.97 | 22.58 | 45.03 | 36.58 |
| Peru | 69 | 55.83 | 19.09 | 50.97 | 26.13 | 52.17 | 24.39 |
| Philippines | 78 | 58.05 | 25.58 | 45.34 | 41.87 | 41.48 | 46.82 |
| Republic of Moldova | 12 | 10.64 | 11.33 | 9.87 | 17.75 | 9.32 | 22.33 |
| Republic of North Macedonia | 3 | 2.61 | 13.00 | 2.32 | 22.67 | 2.15 | 28.33 |
| Rwanda | 259 | 207.81 | 19.76 | 163.79 | 36.76 | 140.30 | 45.83 |
| Saint Lucia | 73 | 66.02 | 9.56 | 60.20 | 17.53 | 48.09 | 34.12 |
| Saint Vincent and the Grenadines | 62 | 56.87 | 8.27 | 49.78 | 19.71 | 32.36 | 47.81 |
| Samoa | 59 | 48.57 | 17.68 | 36.74 | 37.73 | 31.11 | 47.27 |
| Sao Tome and Principe | 146 | 125.63 | 13.95 | 106.46 | 27.08 | 97.03 | 33.54 |
| Senegal | 261 | 225.76 | 13.50 | 181.83 | 30.33 | 142.12 | 45.55 |
| Serbia | 10 | 9.11 | 8.90 | 8.40 | 16.00 | 7.66 | 23.40 |
| Sierra Leone | 443 | 352.61 | 20.40 | 269.39 | 39.19 | 225.64 | 49.07 |
| Solomon Islands | 122 | 97.92 | 19.74 | 76.30 | 37.46 | 54.52 | 55.31 |
| Somalia | 621 | 563.59 | 9.24 | 471.63 | 24.05 | 252.80 | 59.29 |
| South Africa | 127 | 114.99 | 9.46 | 105.30 | 17.09 | 94.33 | 25.72 |
| South Sudan | 1223 | 1116.80 | 8.68 | 951.13 | 22.23 | 569.79 | 53.41 |
| Sri Lanka | 29 | 25.39 | 12.45 | 21.80 | 24.83 | 16.01 | 44.79 |
| Sudan | 270 | 251.18 | 6.97 | 220.93 | 18.17 | 170.84 | 36.73 |
| Suriname | 96 | 80.80 | 15.83 | 73.39 | 23.55 | 68.05 | 29.11 |
| Syrian Arab Republic | 30 | 26.45 | 11.83 | 22.06 | 26.47 | 15.58 | 48.07 |
| Tajikistan | 17 | 13.93 | 18.06 | 10.58 | 37.76 | 8.75 | 48.53 |
| Thailand | 29 | 25.79 | 11.07 | 23.11 | 20.31 | 21.08 | 27.31 |
| Timor-Leste | 204 | 178.44 | 12.53 | 146.86 | 28.01 | 114.23 | 44.00 |
| Togo | 399 | 346.01 | 13.28 | 278.79 | 30.13 | 194.82 | 51.17 |
| Tonga | 126 | 100.55 | 20.20 | 91.88 | 27.08 | 90.86 | 27.89 |
| Tunisia | 37 | 33.03 | 10.73 | 29.63 | 19.92 | 25.26 | 31.73 |
| Turkey | 17 | 15.48 | 8.94 | 14.25 | 16.18 | 13.01 | 23.47 |
| Turkmenistan | 5 | 4.49 | 10.20 | 4.05 | 19.00 | 3.95 | 21.00 |
| Uganda | 284 | 241.01 | 15.14 | 182.63 | 35.69 | 128.08 | 54.90 |
| Ukraine | 17 | 14.70 | 13.53 | 13.37 | 21.35 | 11.80 | 30.59 |
| United Republic of Tanzania | 238 | 185.79 | 21.94 | 118.98 | 50.01 | 97.75 | 58.93 |
| Uzbekistan | 30 | 26.68 | 11.07 | 23.42 | 21.93 | 19.31 | 35.63 |
| Vanuatu | 94 | 71.15 | 24.31 | 53.10 | 43.51 | 46.58 | 50.45 |
| Viet Nam | 46 | 37.80 | 17.83 | 32.96 | 28.35 | 26.51 | 42.37 |
| Yemen | 183 | 155.18 | 15.20 | 114.41 | 37.48 | 64.17 | 64.93 |
| Zambia | 135 | 113.89 | 15.64 | 88.40 | 34.52 | 65.68 | 51.35 |
| Zimbabwe | 357 | 292.97 | 17.94 | 226.42 | 36.58 | 168.38 | 52.83 |

Notes：Maternal mortality ratio (MMR) defined as maternal deaths per 100,000 live births for women of reproductive age (15~49 years). No scale-up (Scenario 0), we assumed that coverage of every health intervention didn’t change from baseline. Modest scale-up (Scenario 1), we assumed that coverage of every health intervention increased 2% per year up to a maximum of 100%. Substantial scale-up (Scenario2), we assumed that coverage of every health intervention increased 5% per year up to a maximum of 100%. Universal coverage (Scenario 3), we assumed that coverage of every health intervention reached 95% by 2030.

**Table S6** **Additional maternal lives saved by 2030 in three scenarios by intervention among 126 LMICs**

| **African Region** | | | |
| --- | --- | --- | --- |
|  | Scenario1:  Modest scale-up | Scenario2:  Substantial scale-up | Scenario3:  Universal coverage |
| **Periconceptual** | **2205** | **5509** | **17250** |
| Safe abortion services | 2205 | 5509 | 17250 |
| **Pregnancy** | **3092** | **7661** | **17918** |
| TT - Tetanus toxoid vaccination | 44 | 64 | 40 |
| Micronutrient supplementation (iron and multiple micronutrients) | 149 | 378 | 811 |
| Hypertensive disorder case management | 2899 | 7219 | 17067 |
| **Childbirth** | **31569** | **66994** | **102482** |
| Clean birth environment | 2344 | 4834 | 6224 |
| MgSO4 for eclampsia | 3532 | 7068 | 8537 |
| Antibiotics for preterm or prolonged PROM | 1083 | 2243 | 3313 |
| Antibiotics for maternal sepsis | 3282 | 6796 | 10037 |
| Assisted vaginal delivery | 2809 | 6804 | 15626 |
| Uterotonics for postpartum hemorrhage | 6575 | 13526 | 15097 |
| Manual removal of placenta | 1662 | 3566 | 6244 |
| Removal of retained products of conception | 1650 | 3560 | 6416 |
| Cesarean delivery | 5038 | 10815 | 14816 |
| Blood transfusion | 3594 | 7782 | 16172 |
| **Region of the Americas** | | | |
|  | Scenario1:  Modest scale-up | Scenario2:  Substantial scale-up | Scenario3:  Universal coverage |
| **Periconceptual** | **59** | **150** | **352** |
| Safe abortion services | 59 | 150 | 352 |
| **Pregnancy** | **91** | **230** | **511** |
| TT - Tetanus toxoid vaccination | 1 | 1 | 0 |
| Micronutrient supplementation (iron and multiple micronutrients) | 1 | 2 | 4 |
| Hypertensive disorder case management | 89 | 227 | 507 |
| **Childbirth** | **743** | **1279** | **1672** |
| Clean birth environment | 47 | 72 | 73 |
| MgSO4 for eclampsia | 134 | 187 | 147 |
| Antibiotics for preterm or prolonged PROM | 24 | 43 | 48 |
| Antibiotics for maternal sepsis | 74 | 131 | 142 |
| Assisted vaginal delivery | 116 | 284 | 518 |
| Uterotonics for postpartum hemorrhage | 134 | 180 | 139 |
| Manual removal of placenta | 31 | 65 | 88 |
| Removal of retained products of conception | 30 | 62 | 103 |
| Cesarean delivery | 87 | 115 | 115 |
| Blood transfusion | 66 | 140 | 299 |
| **South-East Asia Region** | | | |
|  | Scenario1:  Modest scale-up | Scenario2:  Substantial scale-up | Scenario3:  Universal coverage |
| **Periconceptual** | **609** | **1518** | **2999** |
| Safe abortion services | 609 | 1518 | 2999 |
| **Pregnancy** | **560** | **1380** | **3426** |
| TT - Tetanus toxoid vaccination | 11 | 11 | 4 |
| Micronutrient supplementation (iron and multiple micronutrients) | 25 | 63 | 91 |
| Hypertensive disorder case management | 524 | 1306 | 3331 |
| **Childbirth** | **6508** | **11191** | **12837** |
| Clean birth environment | 303 | 562 | 458 |
| MgSO4 for eclampsia | 776 | 1703 | 1412 |
| Antibiotics for preterm or prolonged PROM | 159 | 323 | 305 |
| Antibiotics for maternal sepsis | 484 | 978 | 921 |
| Assisted vaginal delivery | 447 | 1085 | 2518 |
| Uterotonics for postpartum hemorrhage | 1446 | 2448 | 1841 |
| Manual removal of placenta | 272 | 580 | 977 |
| Removal of retained products of conception | 269 | 578 | 991 |
| Cesarean delivery | 1812 | 1778 | 883 |
| Blood transfusion | 540 | 1156 | 2531 |
| **European Region** | | | |
|  | Scenario1:  Modest scale-up | Scenario2:  Substantial scale-up | Scenario3:  Universal coverage |
| **Periconceptual** | **3** | **7** | **10** |
| Safe abortion services | 3 | 7 | 10 |
| **Pregnancy** | **8** | **17** | **39** |
| TT - Tetanus toxoid vaccination | 0 | 0 | 0 |
| Micronutrient supplementation (iron and multiple micronutrients) | 0 | 0 | 0 |
| Hypertensive disorder case management | 8 | 17 | 39 |
| **Childbirth** | **62** | **114** | **154** |
| Clean birth environment | 3 | 4 | 4 |
| MgSO4 for eclampsia | 11 | 18 | 11 |
| Antibiotics for preterm or prolonged PROM | 2 | 3 | 3 |
| Antibiotics for maternal sepsis | 6 | 8 | 7 |
| Assisted vaginal delivery | 14 | 37 | 79 |
| Uterotonics for postpartum hemorrhage | 12 | 16 | 8 |
| Manual removal of placenta | 2 | 5 | 6 |
| Removal of retained products of conception | 2 | 4 | 7 |
| Cesarean delivery | 5 | 7 | 8 |
| Blood transfusion | 5 | 12 | 21 |
| **Eastern Mediterranean Region** | | | |
|  | Scenario1:  Modest scale-up | Scenario2:  Substantial scale-up | Scenario3:  Universal coverage |
| **Periconceptual** | **313** | **784** | **1750** |
| Safe abortion services | 313 | 784 | 1750 |
| **Pregnancy** | **560** | **1389** | **3494** |
| TT - Tetanus toxoid vaccination | 10 | 15 | 14 |
| Micronutrient supplementation (iron and multiple micronutrients) | 21 | 52 | 132 |
| Hypertensive disorder case management | 529 | 1322 | 3348 |
| **Childbirth** | **4390** | **9245** | **13799** |
| Clean birth environment | 253 | 540 | 774 |
| MgSO4 for eclampsia | 706 | 1537 | 1665 |
| Antibiotics for preterm or prolonged PROM | 119 | 249 | 384 |
| Antibiotics for maternal sepsis | 357 | 758 | 1170 |
| Assisted vaginal delivery | 426 | 1035 | 2595 |
| Uterotonics for postpartum hemorrhage | 854 | 1778 | 1854 |
| Manual removal of placenta | 207 | 446 | 764 |
| Removal of retained products of conception | 207 | 444 | 778 |
| Cesarean delivery | 812 | 1489 | 1872 |
| Blood transfusion | 449 | 969 | 1943 |
| **Western Pacific Region** | | | |
|  | Scenario1:  Modest scale-up | Scenario2:  Substantial scale-up | Scenario3:  Universal coverage |
| **Periconceptual** | **60** | **130** | **222** |
| Safe abortion services | 60 | 130 | 222 |
| **Pregnancy** | **77** | **189** | **424** |
| TT - Tetanus toxoid vaccination | 1 | 2 | 3 |
| Micronutrient supplementation (iron and multiple micronutrients) | 1 | 2 | 2 |
| Hypertensive disorder case management | 75 | 185 | 419 |
| **Childbirth** | **1074** | **1831** | **2533** |
| Clean birth environment | 20 | 31 | 30 |
| MgSO4 for eclampsia | 114 | 229 | 153 |
| Antibiotics for preterm or prolonged PROM | 9 | 20 | 20 |
| Antibiotics for maternal sepsis | 32 | 60 | 61 |
| Assisted vaginal delivery | 106 | 250 | 507 |
| Uterotonics for postpartum hemorrhage | 276 | 347 | 252 |
| Manual removal of placenta | 44 | 98 | 160 |
| Removal of retained products of conception | 44 | 97 | 165 |
| Cesarean delivery | 347 | 518 | 797 |
| Blood transfusion | 82 | 181 | 388 |

**Table S7** **Estimated relative reductions of** **MMR by 2030 in three scenarios among LMICs in Region of the Americas and Western Pacific Region**

|  | **No scale-up** | **Substantial scale-up** | | **Universal coverage** | |
| --- | --- | --- | --- | --- | --- |
|  | MMR  (per 100,000 live births) | MMR  (per 100,000 live births) | Reduction  (%) | MMR  (per 100,000 live births) | Reduction  (%) |
| **Region of the Americas** | **87** | **63.09** | **23.56** | **49.83** | **36.84** |
| Argentina | 45 | 38.98 | 13.38 | 37.80 | 16.00 |
| Belize | 130 | 102.39 | 21.24 | 103.66 | 20.26 |
| Bolivia | 161 | 115.04 | 28.55 | 103.28 | 35.85 |
| Brazil | 72 | 60.76 | 15.61 | 58.40 | 18.89 |
| Colombia | 75 | 64.06 | 14.59 | 60.48 | 19.36 |
| Costa Rica | 22 | 19.20 | 12.73 | 20.01 | 9.05 |
| Cuba | 39 | 34.59 | 11.31 | 32.86 | 15.74 |
| Dominican Republic | 107 | 94.71 | 11.49 | 94.83 | 11.37 |
| Ecuador | 66 | 51.52 | 21.94 | 46.38 | 29.73 |
| El Salvador | 43 | 36.32 | 15.53 | 32.67 | 24.02 |
| Grenada | 21 | 16.40 | 21.90 | 9.07 | 56.81 |
| Guatemala | 96 | 72.10 | 24.90 | 59.74 | 37.77 |
| Haiti | 350 | 238.00 | 32.00 | 144.85 | 58.61 |
| Honduras | 72 | 53.16 | 26.17 | 48.03 | 33.29 |
| Jamaica | 99 | 82.27 | 16.90 | 77.97 | 21.24 |
| Mexico | 59 | 49.91 | 15.41 | 47.27 | 19.88 |
| Nicaragua | 78 | 57.74 | 25.97 | 49.24 | 36.87 |
| Paraguay | 71 | 59.39 | 16.35 | 57.60 | 18.87 |
| Peru | 69 | 55.99 | 18.86 | 61.03 | 11.55 |
| Saint Lucia | 73 | 62.21 | 14.78 | 51.09 | 30.01 |
| Saint Vincent and the Grenadines | 62 | 52.47 | 15.37 | 35.73 | 42.37 |
| Suriname | 96 | 76.20 | 20.63 | 75.35 | 21.51 |
| **Western Pacific Region** | **89** | **63.32** | **27.32** | **53.08** | **38.73** |
| Cambodia | 218 | 149.89 | 31.24 | 129.45 | 40.62 |
| China | 23 | 17.17 | 25.35 | 11.81 | 48.65 |
| Fiji | 38 | 30.71 | 19.18 | 26.90 | 29.21 |
| Kiribati | 76 | 48.85 | 35.72 | 41.91 | 44.86 |
| Lao Peope's Democratic Republic | 126 | 93.24 | 26.00 | 75.18 | 40.33 |
| Malaysia | 21 | 17.19 | 18.14 | 14.03 | 33.19 |
| Micronesia (Fed. States of) | 74 | 57.12 | 22.81 | 36.99 | 50.01 |
| Mongolia | 39 | 31.40 | 19.49 | 32.27 | 17.26 |
| Papua New Guinea | 192 | 137.63 | 28.32 | 100.81 | 47.49 |
| Philippines | 78 | 50.42 | 35.36 | 51.90 | 33.46 |
| Samoa | 59 | 39.22 | 33.53 | 33.95 | 42.46 |
| Solomon Islands | 122 | 87.57 | 28.22 | 63.30 | 48.11 |
| Tonga | 126 | 94.25 | 25.20 | 94.18 | 25.25 |
| Vanuatu | 94 | 61.49 | 34.59 | 55.35 | 41.12 |
| Viet Nam | 46 | 33.72 | 26.70 | 28.13 | 38.85 |

Notes：Maternal mortality ratio (MMR) defined as maternal deaths per 100,000 live births for women of reproductive age (15~49 years). No scale-up, we assumed that coverage of every health intervention didn’t change from baseline. Substantial scale-up, we assumed that coverage of every childbirth intervention increased 5% per year up to a maximum of 100%. Universal coverage, we assumed that coverage of every childbirth intervention reached 95% by 2030.
